# Conservative Versus Surgical Management of Elbow Medial Ulnar Collateral Ligament Injury: A Systematic Review

**DOI:** 10.1111/os.12571

**Published:** 2019-11-26

**Authors:** Carlo Biz, Alberto Crimì, Elisa Belluzzi, Nicola Maschio, Riccardo Baracco, Andrea Volpin, Pietro Ruggieri

**Affiliations:** ^1^ Orthopaedic Clinic, Department of Surgery, Oncology and Gastroenterology University of Padova Padova Italy; ^2^ Musculoskeletal Pathology and Oncology Laboratory, Department of Surgery, Oncology and Gastroenterology University of Padova Padova Italy; ^3^ Trauma and Orthopaedic Department Royal Derby Hospital NHS Foundation Trust Derby UK

**Keywords:** Ligament injury, Elbow instability, Elbow dislocation, Sport injuries

## Abstract

**Objective:**

Several studies have been published regarding the treatment of medial ulnar collateral ligament (MUCL) injuries for professional overhead athletes. However, there is a paucity of data regarding non‐professional athletes. The aim of this systematic review was to compare the rate of outcome scores and complications of conservative versus operative treatments both in non‐professional athletes and in non‐sport‐related trauma patients with MUCL lesions.

**Methods:**

A systematic review of the published literature was performed by applying the PRISMA guidelines. A search was conducted using three databases: Medline, Science Direct, and Web of Science. The keywords used were “ulnar collateral ligament injury,” “elbow,” “surgery,” and “conservative treatment”. Patients were divided into three groups: patients who underwent *conservative treatment* (C‐group), *surgical treatment* (S‐group), and *surgery after a failed conservative treatment* (C&S‐group). Clinical outcomes were analyzed: Disability of Arm, Shoulder and Hand (DASH), Conway scale, Carson score, and Kerlan–Jobe Orthopaedic Clinic score (KJOC).

**Results:**

A total of 15 studies were included, evaluating 513 patients. Although good and excellent outcomes were found for most patients during daily and/or sport activities, independently of the type of treatment, the C‐group had better results. Excellent results were found in 98.8% of the C‐group, in 88.1% of the S‐group, and in 87.7% of the C&S‐group. The complication rate in the C‐group was statistically higher compared to the S and C&S groups (*P* < 0.001). However, its complication rate was higher with lower patient satisfaction.

**Conclusions:**

There is insufficient evidence to establish statistically significant differences in the effects of conservative versus surgical treatments on the functional outcomes of patients with MUCL lesions. However, a period of rehabilitation therapy and the functional request of the single injured subject are useful to discern which patients genuinely require MUCL surgical repair.

## Introduction

In contrast to the normal activities of daily living (ADL), which generally do not cause significant valgus stress on the medial side of the elbow, overhead sports can put tremendous stress^1^, ^2^ on this joint, whose main static anatomical stabilizer is the medial ulnar collateral ligament (MUCL)^3^, ^4^. This ligament, running from the distal part of the medial epicondyle to the sublime tubercle of the medial ulna^5^, is composed of three different bundles: anterior oblique (the most significant stabilizer to valgus stress), posterior oblique, and transverse^6^. MUCL insufficiency is mainly described as a chronic progressive lesion, rather than an acute lesion, the former being a consequence of repetitive trauma, which more frequently affects young athletes practicing sports such as baseball, javelin throwing, gymnastics, wrestling, football, and tennis^7^. However, it can also be a consequence of an acute rupture after a traumatic elbow dislocation, often in association with articular fractures^6^.

The description of these consequences, due to an MUCL injury, was first reported by Waris in 1946^8^. Over the past decade, with the progressive understanding of anatomy, looking at the etiopathogenesis and biomechanics of this lesion, we have come to understand that elbow joint instability results in constant pain, poor elbow function with loss of extension, decreased performance, and subsequent joint degeneration. In contrast, medial swelling with point tenderness over the course of the ligament and without limited range of motion (ROM) is more commonly detected in acute or subacute cases^4^. The tests for clinical instability include the moving valgus test^9^, the valgus stress test at 30°, 60° and 90°, the Milk test, and the valgus extension overload test^6^. Even the integrity of the ulnar nerve should be carefully verified during the clinical examination.

Radiological images are essential for the diagnosis. In particular, standard antero–posterior and lateral radiograph views are usually normal, even if in complicated cases they can show avulsion of ulnar sublime tubercle, soft tissue swelling, or other associated conditions such as loose bodies, chondral defects or osteophytes and subsequently heterotopic ossifications (HO)^6^. MRI can also highlight a full thickness lesion of the MUCL, although the gold standard for partial thickness lesions is an arthro‐MRI^6^. A CT arthrogram remains a possible option in patients who cannot undergo MRI for clinical reasons or in cases of associated fractures for preoperative planning. Moreover, static and dynamic ultrasound are also used, as reported by Ciccotti et al., in the diagnosis of MUCL ligament injuries, even if the soft tissue damage definition is superior in MRI images^10^.

It is clearly accepted that MUCL insufficiency requires treatment to restore medial elbow stability and its complete function in ADL as well as during sports practice. Conservative treatment consists of a sequential and progressive multiphase approach with overlapping stages in order to gain both the quickest and the best clinical outcomes. In a nonoperative rehabilitation program, a wide ROM (30°–110°) is permitted even in the first phase just after a few days of rest and isometric exercises, usually with a brace to prevent adjunctive valgus loading on the elbow. In addition, ice and anti‐inflammatory medications are prescribed to decrease pain. In the second phase, there is an increase of allowed ROM between 5° to 10° degrees per week until pain‐free full ROM is achieved by the fourth week, ever avoiding elbow valgus loading. Stretching and isotonic exercises are encouraged to develop dynamic stabilization and proprioceptive control of the elbow. The third phase starts at approximately 6–7 weeks post‐injury, increasing general upper arm strength and resistance, including using plyometric exercises to regain complete pre‐injury elbow function. Once the patient has regained painless full ROM, arm strength and elbow stability, they are allowed to proceed with the fourth phase: the progressive return to usual sport or daily activities^11^. Certainly, although following precise rehabilitation protocols, the outcomes can be unpredictable. Rettig et al. showed that following nonoperative treatment only 42% of athletes returned to sport activity after 6 months^12^. Another recent conservative option is the platelet‐rich plasma (PRP) injection, as described by Podesta et al., providing positive results for partial MUCL tears^13^, as well as bracing and therapy, as discussed by Savoie et al., leading to MUCL healing^6^. Moreover, the application of a dynamic external fixator for a few weeks has shown good outcomes, allowing the patient to start motion exercises immediately^4^.

Several different techniques using reconstruction and repair have been described^14^, ^15^. The use of suture anchors for proximal or distal avulsion lesions has been tested for both acute and chronic lesions^16^, ^17^; direct repair remains a viable option for mid‐part disruption, while reconstruction is possible using ipsilateral palmaris longus, gracilis, toe extensors, allographs, or flexor‐pronator fascia patches^6^, ^18^.

While the improved surgical options have, indeed, translated to improved functional and patient outcomes, the treatment algorithm for MUCL tears remains elusive^15^, ^19^. Hurwit et al. surveyed 159 members of the American Shoulder and Elbow Surgeons and reported that professional athletes and those with complete tears were indicated for surgery by consensus^20^; opinion was more divided on how to treat partial tears or non‐professional athletes^20^. It is well known that operative treatment is the gold standard for professional athletes. Several systematic reviews and meta‐analyses analyzing return to sport after surgery have been published^6^, ^15^, ^18^, ^21^, ^22^. However, no reviews have focused on understanding which treatment, conservative or surgical, is the best option for low‐function‐demand patients.

Although there is a growing volume of knowledge in the literature regarding the treatment of MUCL injuries for professional overhead athletes^22^, there is a paucity of data regarding non‐professional athletes. Hence, the purpose of this systematic review was to compile the current literature on MUCL injuries both in non‐professional athletes and in non‐sport‐related trauma patients, to compare the different management treatments.

## Material and Methods

### *Search Strategy*

The present review and its procedures were organized, conducted, and reported following the Preferred Reporting Items for Systematic Reviews and Meta‐Analyses (PRISMA) guidelines with a PRISMA checklist and algorithm^23^, ^24^. A medical librarian‐assisted electronic search was conducted using three different databases: Medline, Science direct, and Web of Science. In each database an advanced search was conducted using the following combination of free terms (ulnar collateral ligament injury) and MESH terms (elbow and surgery): (i) “ulnar collateral ligament injury” AND “elbow” AND “surgery”; (ii) “ulnar collateral ligament injury” AND “elbow” AND “conservative treatment”.

### *Selection Criteria*

A 10‐year time selection, from January 2007 to May 2018, was used. Only studies in English of all levels of evidence on humans, published in peer‐reviewed journals, were included. Articles were considered eligible if they met the following PICOS criteria:

- Population: a target population consisting of young and adult patients, with MUCL rupture, partial or total, resulting in medial elbow instability, simplex or complex, which occurred during sport or non‐sport related activities;
- Intervention and comparisons: the treatment applied based on current conservative or surgical protocols for these injuries, or surgery after failure of a conservative treatment;
- Outcomes: the outcomes reported according to validated international assessment tools (Disability of Arm, Shoulder and Hand [DASH], Conway scale, Carson score, Kerlan–Jobe Orthopaedic Clinic score [KJOC]).

Exclusion criteria: (i) research articles enrolling a large series of professional athletes were excluded; and (ii) expert opinion articles, editorials, letters to the editor, unpublished series, biomechanical reports, studies on animals, cadavers, in vitro or animal studies, case reports, literature reviews, technical notes, and instructional courses. All articles relevant to the subject were retrieved, and their bibliographies hand‐searched for further references in this context.

### *Selection Method*

Two reviewers independently screened the title and reviewed the abstract and biographies of each publication for applicability per inclusion/exclusion criteria and additional relevant articles up to June 2018. A close reading of all papers and extracted data was then performed to minimize selection bias and errors. A cross‐reference research of the selected articles was also performed to obtain other relevant articles for the study. The selection was based on the abstract's content: if inclusion or exclusion of the article was not possible based on the abstract, the full‐text article was read. Full‐text studies identified by either author as potentially relevant were acquired for further review by both authors. Moreover, the lists of references of the full‐text studies were also reviewed to look for other potential articles eligible for inclusion. Finally, to avoid bias, the selected articles, the relative list of references, and the articles excluded from the study were reviewed, assessed, and discussed by all the authors. If there was disagreement among investigators regarding the inclusion and exclusion criteria, the senior investigator made the final decision.

### *Quality Assessment*

To assess the quality of the selected studies, a Downs and Black scale was used^25^. It assesses methodology using 27 items with a range 0 to 28 points, divided into five sections (Reporting, External validity, Internal validity – bias, Internal validity – confounding or selection bias, and Power). This scale evaluates methodologies and it is also indicated for case series. The study methodology is considered excellent (26–28 points), good (20–25 points), fair (15–19 points), or poor (<14 points).

### *Data Extraction and Elaboration*

After downloading the PDF files of each study included in the selection, the data were extracted into a customized database for the subsequent analysis following *this data extraction form*:

- *Study design and level of evidence*.
- *Population*: Sample size, characteristics (non‐professional athletes or non‐sport‐related trauma patients), quality elements (type of trauma), patients’ demographic characteristics (e.g., age, sex, dominant side, and competition level), and length of follow‐up.
- *Type of treatment*: Surgery, conservative treatment, and surgery after failure of conservative treatment.
- *Intra and postoperative complications*: Technical error, re‐rupture, re‐surgery, ulnar nerve persistent symptoms, post‐treatment varus‐valgus instability, heterotopic ossifications, and ongoing elbow pain stiffness during sport/life activities.
- Clinical outcomes: Disability of Arm, Shoulder and Hand (DASH); Conway scale; Carson score; Kerlan–Jobe Orthopaedic Clinic score (KJOC); Andrews‐Timmerman score; Mayo elbow performance score (MEPS); return to work/play 1 year after surgery.

### *Statistical Analysis*

Descriptive statistics of the eligible studies were performed. Continuous variable data (age and follow‐up time) were reported as mean and range. Categorical variable data (gender, dominant side, and clinical outcomes) were reported as frequencies with percentages. The Kruskal–Wallis test, with Bonferroni post‐hoc tests, was used to analyze categorical data. A *P‐value* <0.05 was considered as statistically significant. All analyses were performed with IBM SPSS Statistics for Windows, Version 25.0 (IBM, Armonk, NY, USA). A meta‐analysis was not possible to perform because there was no intra‐study comparison data as the studies had different patient‐reported outcome measures.

## Results

### *Literature Search*

**Figure 1:**
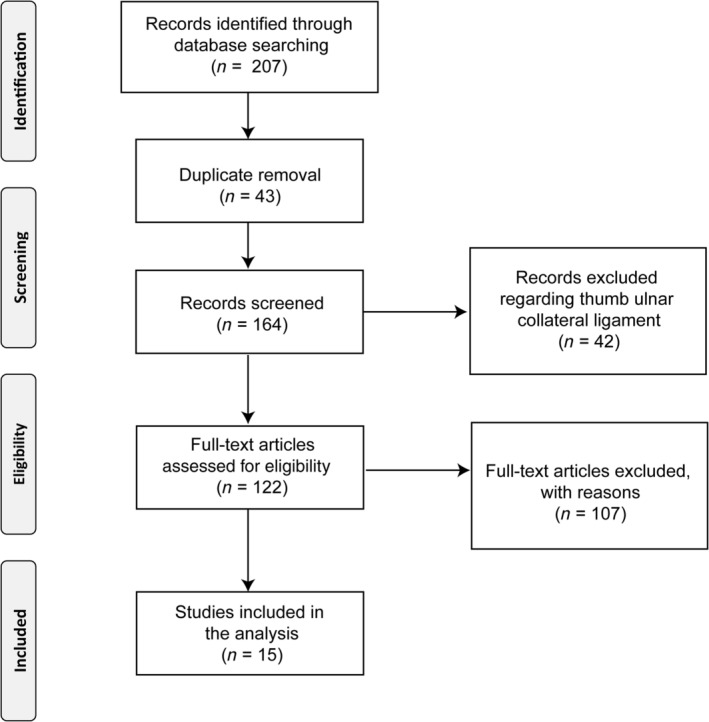
Preferred Reporting Items for Systematic Reviews and Meta‐Analyses (PRISMA): Flow chart diagram for inclusion and exclusion of paper process. For this study, 122 articles were assessed for eligibility after screening; among these, 15 papers fulfilled the selection criteria and were included in the analysis.

The literature search obtained 95 papers from Medline, 97 papers from Web of Science, and 15 papers from Science Direct. A total of 164 articles were initially identified by the search. Papers (42) on the thumb ulnar collateral ligament papers were immediately excluded. Of the remaining 122 manuscripts initially selected based on the search strategy of this study, 15 relevant reports were finally identified (Table 1 and Fig. 1). The outcomes of conservative and surgical treatments for rupture of the MUCL, utilizing diverse techniques, were compared in the 15 selected studies from January 2007 to May 2018. Preoperative and postoperative outcomes for each study were analyzed (Table 1). Level of evidence (I, II, III, or IV) was reported for the included studies (Table 1).

**Table 1:** Demographics, level of evidence, type of intervention, and outcomes of included studies

First author (year)	Type of study	Evidence level	D&B	Patients (M/F)	Mean age years (range)	Treatment	Procedure	Trauma	Patients	Follow up (months)	Outcome Evaluation
Iordens et al.^26^	Case series	IV	15	27 (13/14)	52 (38–59)	C	External Fixation	19 LE 8 HE	—	12	DASH, MEPS
Kesmezacar et al.^27^	Case series	IV	12	21 (16/5)	35 (16–59)	C	Closed Reduction/ plaster splint/ hinged brace	—	—	34	MEPS
Dines et al.^28^	Case series	IV	12	22	20.1 (16–24)	S	DANE TJ	—	Athletes	36	Conway Scale
Dines et al.^29^	Case Series	IV	9	15 (15/0)	20.1 (19–30)	S	Docking technique	—	Athletes	24	Conway Scale
Erickson et al.^30^	Case series	IV	12	187	19.6	S	Docking technique	—	Athletes	60 (of 85 patients)	Conway Scale, KJOC
Osbahr et al.^31^	Case series	IV	15	8	33.4	S	Docking technique	—	Older athletes	/	Modified Conway scale
Rhyou et al.^32^	Retrospective cohort	III	15	29 (15/14)	37 (18–57)	S	Suture anchors	General Trauma	—	32 (26 patients completed the follow up)	DASH, MEPS
Richard et al.^33^	Case series	IV	9	11	—	S	Suture and drill holes (considered open repair)	—	Athletes	16	DASH
Adolfsson et al.^34^	Case series	IV	9	8 (1/7)	54 (30–86)	C&S	Closed reduction and immobilization in a plaster splint + open surgical repair	6 LE 2 HE	—	24–52	Modified MEPS
Chen et al.^18^	Case series	IV	12	9 (6/3)	34 (13–52)	C&S	Reconstruction with autografts External fixator, rehabilitation + suture anchors	7 LE 2 HE	—	19.6	MEPS
Dines et al.^7^	Case series	IV	12	10	18.5 (18–21)	C&S	Rest, physical therapy + docking technique	—	Athletes	28.9	Conway Scale
Jones et al.^35^	Case Series	IV	9	55 (51/4)	17.4 (15.9–19)	C&S	Rest, physical therapy + docking technique	—	Athletes	24	Conway Scale
Kodde et al.^36^	Case series	IV	13	20 (7/13)	22 8(18–35)	C&S	Physical therapy + interference screw technique	—	Athletes	55	Conway Scale
Podesta et al.^13^	Case series	IV	14	34 (28/6)	18 (14–34)	C&1/34 S	PRP injection and physical therapy + 1 patient open ligament reconstruction	—	Athletes	18	DASH
Savoie et al.^37^	Case series	IV	9	60 (47/13)	17.2 (14.8–22)	C&S	Rehabilitation + suture anchors	—	Athletes	59.2	Andrews‐ Carson score

C, conservative treatment; C&S, failure of conservative treatment followed by surgery; DASH, Disability of Arm, Shoulder and Hand; D&B, Downs and Black score; HE, high‐energy; KJOC, Kerlan–Jobe Orthopaedic Clinic score; LE, low‐energy; Pts, patients; S, surgery treatment.

### *Quality Assessment*

The main value of the Downs and Black scale was 11.1 points, showing that the mean quality of included studies was poor. In fact, among the 15 studies considered, 12 had poor results^7^, ^13^, ^18^, ^27^, ^28^, ^29^, ^30^, ^33^, ^34^, ^35^, ^36^, ^37^ and 3 had fair results^26^, ^31^, ^32^ (5 papers had 9 points^29^, ^33^, ^34^, ^35^, ^37^, 5 papers had 12 points^7^, ^18^, ^27^, ^28^, ^30^, 1 paper had 13 points^36^, 1 paper had 14 points^13^, and 3 papers had 15 points^26^, ^31^, ^32^).

### *Patient Population*

The 15 studies included reported on a total of 516 patients; however, 3 patients dropped out of the follow‐up. Therefore, a total of 513 patients were considered, corresponding to 514 elbows. The average age at treatment was 28.45 years, ranging from 13 to 86 years^18^, ^34^. The total number of patients for each study ranged between 8 and 187^30^, ^31^. The elbow involved in the MUCL lesion was specified in only 5 studies: the dominant side was involved in 302 (97.4%) of 310 elbows, while the non‐dominant side was involved in 8 (2.6%) elbows. The type of injury was described in all studies except for 1 study^27^: sport‐related^7^, ^13^, ^28^, ^29^, ^30^, ^31^, ^33^, ^35^, ^36^, ^37^ and accidental trauma‐related MUCL injuries (usually not isolated lesions)^18^, ^26^, ^32^, ^34^ were the two main causes. The types of lesion are detailed in Table 2. Patients were assessed at an average follow‐up period of 32.6 months, ranging from 12 to 60 months.^26^, ^30^ After dividing the patients into three groups according to the different therapeutic treatment: 81 patients had a conservative treatment (C‐group), 269 patients had surgical treatment only (S‐group), and 163 patients underwent surgery after a failed conservative treatment (C&S‐group). In 1 study patients underwent revision surgery for MUCL re‐tear and in another 3 patients had undergone previous MUCL reconstruction^28^, ^29^. Both studies were included in the S group. The mean age for the *C‐group* was 35 years (range 13.9–59 years), the mean age for the S‐group was 26 years (range 15–57 years), and, finally, for the C&S‐group, the mean age was 25.87 years (range 14–86 years).

**Table 2:** Detailed description of treatment and MUCL lesion type

First author (year)	Number of patients	Type of lesion	Type of treatment
Iordens et al.^26^	27	Complex elbow dislocation	Hinged external fixator with no open surgical repair of MUCL for 6 weeks
Kesmezacar et al.^27^	21	Simple elbow dislocation	Closed reduction, plaster splint (4 pts), for 3 weeks, or hinged brace (17 pts); after 1 week active and passive motions started, mean brace use was 27 days (all patients with elbow posterior dislocation)
Dines et al.^28^	22	MUCL insufficiency	DANE TJ technique
Dines et al.^29^	15	MUCL re‐tear	Docking technique
Erickson et al.^30^	187	MUCL rupture	‐ MUCL reconstruction docking technique (110 pts), double‐docking technique (78 pts). ‐ Ipsilateral palmaris longus graft (110 pts) and hamstring autograft (48 pts). ‐ 79 patients needed ulnar nerve transposition (preoperative neurologic symptoms)
Osbahr et al.^31^	8	MUCL insufficiency and flexor‐pronator injury	MUCL reconstruction with docking technique; the flexor‐pronator injury was treated with debridement if tendinotic or reattachment if torn.
Rhyou et al.^32^	29	MUCL insufficiency (21 pts had complete UCL rupture, 8 partial) in patients with displaced radial head and neck fractures; 11 pts also had coronoid fractures.	MUCL repaired and reattached to the original attachment site through a medial approach using a suture anchor
Richard et al.^33^	11	Acute rupture MUCL	MUCL repair with suture + reattachment to bone with drill holes
Adolfsson et al.^34^	8	Simple elbow dislocation	Closed reduction and immobilization in a plaster splint, followed by open surgical repair
Chen et al.^18^	9	MUCL complete rupture	Open reduction–internal fixation with or without cast immobilization in 5 patients, manual reduction and cast immobilization in 3, and Chinese medical adhesive plaster in 1. Moreover, reconstruction with flexor‐pronator fascia patch was attempted with external fixator (after a trial of rehabilitation)
Dines et al.^7^	10	MUCL insufficiency	Failed a course of nonoperative management that included rest, physical therapy, and a structured rehabilitation regimen + MUCL reconstruction with docking technique
Jones et al.^35^	55	MUCL tears	Rest, non‐steroidal anti‐inflammatory medications, and a structured rehabilitation program + MUCL reconstruction with docking technique
Kodde et al.^36^	20	MUCL insufficiency	3 months of physical therapy + open MUCL reconstruction with interference screw technique
Podesta et al.^13^	34	Partial MUCL lesions	Single PRP injection at the MUCL under ultrasound guidance and physical therapy ‐ Open ligament reconstruction in 1 patient (31 weeks after PRP injection)
Savoie et al.^37^	60	MUCL insufficiency	Rest, rehabilitation, bracing, and medication with (10 patients) or without (50 patients) intra‐articular steroid injections + MUCL suture plication with repair to bone with drill holes (9 patients) MUCL suture repair to bone using anchors (51 patients)

MUCL, medial ulnar collateral ligament; PRP, platelet‐rich plasma; pts, patients.

### *Type of Treatment*

For the selected studies, different conservative approaches and surgical treatments were analyzed (Table 2). The first treatments were: hinged external fixator^26^, conservative treatment after closed reduction and short‐term immobilization^27^, PRP injection, and physical therapy^13^. The second treatments were mainly represented (13/15 studies) by ligament open repair procedures, performed using different techniques^7^, ^13^, ^18^, ^28^, ^29^, ^31^, ^32^, ^33^, ^34^, ^35^, ^36^, ^37^. It is worth noting that surgical treatment was performed in 7 studies (C&S‐group) after a failed conservative treatment in 163 patients (31.8% of overall patients)^7^, ^13^, ^18^, ^34^, ^35^, ^36^, ^37^ (Tables 1, 2). In these studies, a conservative treatment (immobilization, physical therapy, and infiltrations) was attempted for a minimum of 3 months^36^ to a maximum of 9 months^35^ before surgery. In particular, the surgical recurrence rate varied according to the different conservative approach. There was a 100% recurrence (8 patients/8 patients) in patients treated with immobilization only, 88.13% (156 patients/177 patients) with physical therapy and 2.9% of the patients (1 patients/34 patients) with infiltrations. The reason for the conservative treatment failure and consequent surgical indication was mainly residual elbow instability associated with inability to perform high‐functional activities. Overall, in C&S and S‐groups, the surgical techniques used were: open repair with sutures, screw fixation on the ulna with docking of the graft on the humeral side (DANE TJ technique), diagnostic arthroscopy and open repair with anchors or direct suture, open reconstruction, and docking techniques.

As reported in Table 2, in the S‐group the surgical procedures were: DANE TJ technique^28^, ^36^, docking and double docking technique^7^, ^30^, ^31^, ^35^, suture anchor^18^, ^32^, ^37^, and suture to bone with drill holes^33^.

### *Clinical Outcome Measurements*

#### *Overall Analysis of Clinical Scores*

Although the studies included in this review were conducted using different outcome evaluation systems, overall clinical improvement of the patients after both conservative and surgical treatments was reported. Most frequently, the clinical and functional outcomes were evaluated using the Conway scale, which was used in 7 studies (46.7%), showing excellent results in 280/317 (88%) of included patients. The other scoring systems used in the reports considered were: DASH in 4 studies (26.7%); MEPS in another 4 (26.7%); KJOC was used in 2 studies (13.3%), while the Carson score and the Andrews–Timmerman score were each only used once (6.7%); the return to sport within 1 year was used as an evaluation criterion in 2 studies (13%). In the 2 conservative‐only treatment papers, the outcomes were evaluated by MEPS, reaching a mean score of 98.45, but only the performance in ADL and not in strenuous physical activities was considered^26^, ^27^.

#### *Comparison of Clinical Scores Among the Different Studies*

Because the clinical outcomes were evaluated using different clinical scores, with several authors assessing their subjects in the selected studies, all patients were divided into four groups to compare the results (if possible), according to the scores reported in each study analyzed: excellent, good, moderate/fair, and poor. In this way, following the original guidelines of each score described in the papers included, the patients reporting a score indicated as excellent, were included in the “excellent group,” patients with a score reported as good, were included in the “good group,” and so on.

In the C‐group, 80 patients reported excellent results and 1 moderate^26^, ^27^. In the S‐group, 237 patients reported excellent, 4 good, 17 moderate/fair, and 11 poor results^28^, ^29^, ^30^, ^31^. In the C&S‐group, 136 patients reported excellent, 7 good, 6 moderate/fair, and 6 poor results^7^, ^35^, ^36^, ^37^. Hence, excellent results were obtained in 98.8% of the C‐group, in 88.1% of the S‐group, and in 87.7% of the C&S‐group, respectively. Comparing the 3 patients’ groups there was a statistical difference (*P* = 0.015). In particular, the C‐group attained better outcomes compared to the other groups (*P* = 0.017 compared to the S‐group; *P* = 0.031 compared to the C&S‐group, respectively).

### *Complications*

Taking the complications into consideration, only post‐treatment complications were considered for the C‐group, while for the S‐group and the C&S‐group, intraoperative complications were differentiated from the postoperative complications. In the first group, the complication rate was 40.1%, when not considering HO; if HO were considered then the complication rate reached 74%. In 2 studies on MUCL rupture in elbow dislocations, the most represented complications were 27 cases of HO (25.7% of all complications reported in the series considered).

It is worth noting that intraoperative complications were not reported for the surgically treated patients (S‐group), while the postoperative complications were recorded in 21 out of 269 patients, a 7.8% rate of complications. In the C&S group, the complication rate was 14.1% (23 patients out of 163). The most represented complications overall in the S‐group and the C&S group were: 28 cases (26.7% of all complications) of ulnar nerve neuritis or ulnar nerve neuropathy, and 8 cases (7.5%) of re‐rupture. There was a statistical significance of *P* < 0.001 when comparing the complication rate between the groups. In particular, the complication rate in the C‐group was statistically higher compared to the S and C&S groups (*P* < 0.001). A detailed description of the complications in each study is reported in the supplementary materials (Table S1).

## Discussion

Elbow MUCL injury still represents a difficult challenge in the orthopaedic context. Therefore, the focus of this study was to analyze, through a review of the current literature, the different methods used to treat MUCL injuries and to find the best evidence to support the effectiveness of either nonoperative or operative treatment of these lesions. The purpose of this systematic review was to compile the current literature on MUCL injuries, both in non‐professional athletes and in non‐sport‐related trauma patients, to understand if there is a consensus on surgically repairing these lesions or treating them conservatively.

To the best of our knowledge, this is the first systematic review analyzing a homogeneous selection of papers published in English, avoiding large series of professional athletes, such as professional baseball players, and including trauma patients with non‐sport‐related MUCL injuries who are often not high‐function‐demand patients. In the 15 studies included, surgical treatment was the most common treatment (S‐group)^28^, ^29^, ^30^, ^31^, ^32^, ^33^ versus 2 studies that exclusively discussed conservative treatment (C‐group)^26^, ^27^; a nonoperative treatment was attempted before surgery in at least 7 papers (C&S‐group)^7^, ^13^, ^18^, ^34^, ^35^, ^36^, ^37^. As these studies were predominantly retrospective (level evidence IV), it is not possible to conclusively identify the best solution for the management of MUCL lesions. Nevertheless, in the literature, a high percentage of satisfactory outcomes, good elbow stability, and a generally low complication rate were reported when surgical treatment was performed^6^.

The most important finding of this review was that good to excellent clinical outcomes were found in most patients, independently of the type of treatment used. Because different clinical scores were used, and some of the studies analyzed did not use a validated outcome measure, we converted the results of single studies for the four possible outcomes scores (poor, fair, good, excellent) to make an estimate accurate enough to represent the sample size and to evaluate if there was a significant difference among the groups. This analysis showed that the clinical outcomes achieved by C‐group were better when compared to the other two. However, the complication rate of this group was higher compared to the others. In particular, the patients treated with a nonoperative approach exhibited the highest complication rate of 74%. In contrast, patients of the S‐group had a complication rate of 7.7% and those of the C&S group showed a complication rate of 14.1%. Overall, considering the entire cohort of included patients (C‐group, S‐group, and C&S‐group), there was a complication rate of 20%. In the S‐group and the C&S‐group there were fewer HO compared to the C‐group (where they are the most represented complications), while a higher rate ‘of postoperative nerve complications was highlighted in the S and C&S groups.

Our systematic review revealed that there were both several advantages and disadvantages associated with conservative and surgical treatments. The results suggest that a brief period of rehabilitation therapy, at least 3 months, is useful to discern which patients really need MUCL repair^12^, ^36^, ^38^, ^39^. Based on the type of the injury (sport‐related or occasional trauma‐related), several options were proposed for nonoperative treatment, such as PRP injection, short‐term immobilization using casting, bracing, or elbow external fixation^26^, ^27^. However, for most patients who do not meet strict surgical indications, the management algorithm and outcomes of nonoperative treatment remain unclear and future randomized control studies are needed.

The surgical treatment preferred was the open reconstruction with different techniques as described in the Results section^28^, ^29^, ^30^, ^31^, ^32^, ^33^. Among these, the most commonly used are: docking technique, suture anchors, and open repair. The procedures used seem to have comparable and overall excellent results. However, the choice of technique performed seems limited to the personal preference of the surgeon. In the S‐group and the C&S‐group, a lower complication rate was found for the docking technique (7.3%), compared to 15.6% of suture anchors and almost 20% of open repair, although the docking technique demonstrated a higher rate of reoperation (3.8%) compared to other techniques. This could be due to the fact that the follow up was not long enough to highlight recurrences in these series and also to the sample selection of patients, excluding professional athletes, who undergo these surgical procedures more frequently. Interestingly, Watson et al. compared the clinical results of patients treated with different surgical techniques in a systematic review including 21 studies and 1368 patients^19^. These authors reported excellent results in 78.9% of patients and excellent results in each surgical technique used (Classic Jobe, modified Jobe, interference screw, docking, and modified docking). In contrast, the aim of our review was not to compare the effectiveness of single surgical procedures, and the analysis of the results in relation to each surgical procedure used would not be reliable because of the limited number of patients who underwent each technique.

Surgical treatment was represented by open reconstruction in 13 studies and the results, even if investigated with different outcome scores, were excellent in 88% of cases according to the Conway scale. Furthermore, the general satisfaction of the patients after surgical treatment was higher compared to the studies with only conservative treatment. In the 2 studies where only a nonoperative treatment was managed, there were excellent MEPS and DASH scores in more than 90% of cases considering only ADL. However, the satisfaction rate was 19% when challenging sports activities were taken into consideration. It has to be considered that MEPS evaluates the elbow function in ADL, while the Conway score is used for athletes’ outcomes^3^. The poorest outcomes in surgically treated patients are reported in Osbahr et al., probably due to the particular cohort of patients included: older players with combined flexor‐pronator and ulnar collateral ligament injuries^31^. When counseling patients with MUCL injuries, it is important to understand their functional requirements. As shown in the Results section, excellent results can be achieved in ADL with a purely conservative treatment. Therefore, it is important to take a detailed history and perform a clinical examination of the patient before discussing the management options.

This review is limited by the level of evidence of the studies included, consequently presenting some drawbacks: first, the studies included are not homogenous for management techniques and outcome scores. In particular, the comparison of outcome results due to the wide variety of scores used and the quality of the included studies made it difficult to undertake a full and statistically reliable comparison of the published data. The poor homogeneity of clinical evaluation scores has played an important role in relation to the low possibility of obtaining a precise comparison, not only between conservative and surgical treatment but also among various types of open repair or reconstruction. Unfortunately, our selection included mainly case series, case‐control studies, or retrospective cohort papers with small sample size, lack of control groups, and restricted statistical comparison. Hence, most studies in the series had low levels of evidence (III or IV): 14 papers had a level of evidence IV, while only 1 had a level of evidence III.

Some of the studies exhibited selection bias, 1 study did not report outcome results, and others did not include some of the complications in the final results^7^, ^32^, ^34^. The stated complication rate, extracted from retrospective data, was likely to be an underestimation of the true complication rate, because the authors of the analyzed studies may not have reported minor complications despite their occurrence. In addition, as the aim of the study was to analyze the outcome of MUCL injuries in non‐high‐function‐demand patients, the age range was wider (13–86 years) with respect to those described by reviews regarding the same injuries in professional athletes. However, the median age in most of the included studies was around 17–37 years old, which is in line with epidemiologic data already reported^40^. There were only two studies reporting a mean age of 52 years (range, 38–59 years) and 54 (30–86) years, respectively. In particular, in the second study, only one 86‐year‐old patient and two over 60 years old were included, reporting good clinical and functional outcomes regardless of the treatment adopted.

Moreover, the activity (level of sport or work activities) of the different patients was very heterogeneous. Finally, longer follow up and randomized controlled studies are necessary to guide future indications for treatment of MUCL injuries. Certainly, level I or level II studies are necessary to understand what the best treatment option for the management of UCL injuries is, and standardized methods of functional outcomes assessment are necessary to improve knowledge concerning functional results of MUCL repair.

### *Conclusion*

**Figure 2:**
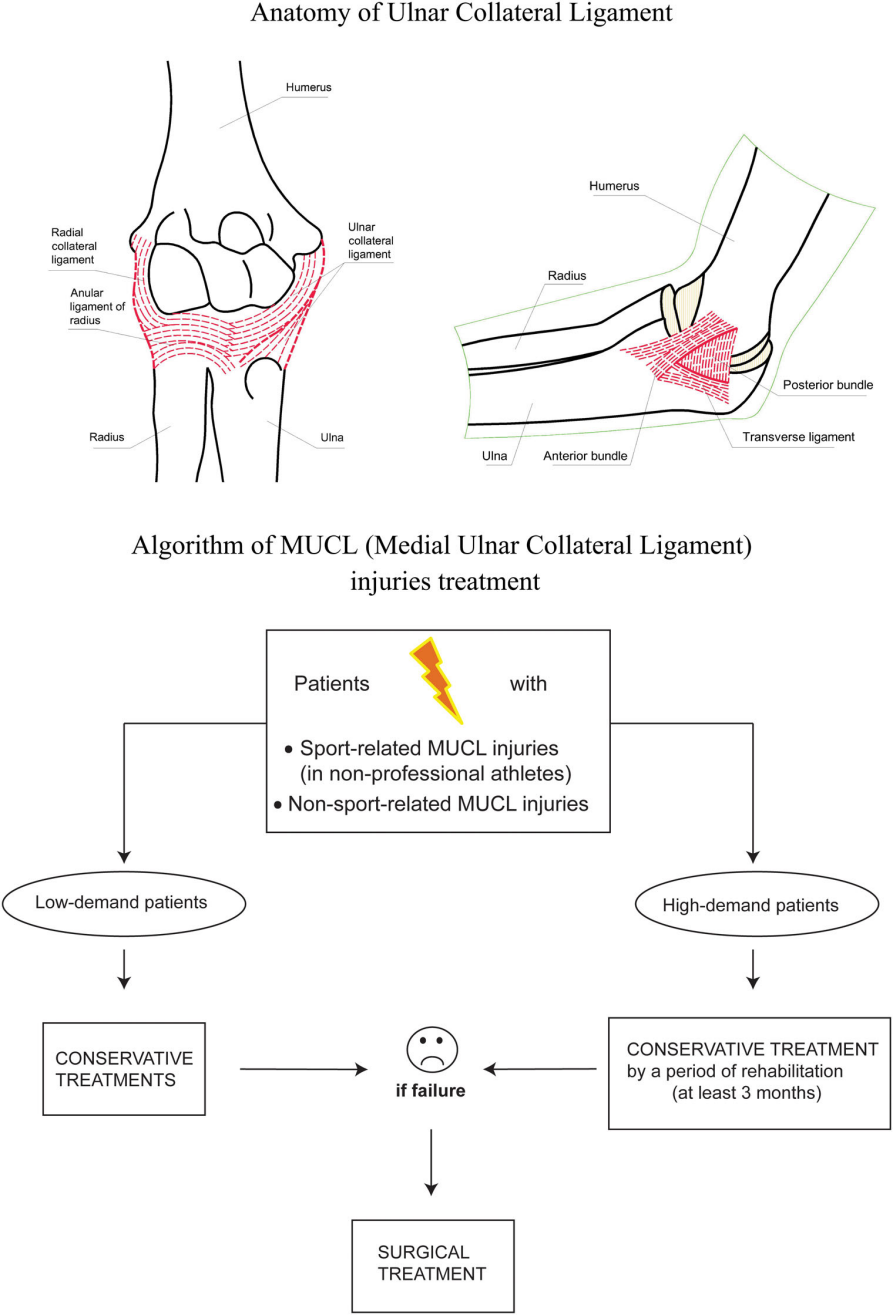
Algorithm of medial ulnar collateral ligament (MUCL) injury treatment. According to our findings, at first both patient groups (low‐function‐demand and high‐function‐demand) should be treated conservatively. Whether residual elbow instability persists, surgery is indicated for both groups, regardless of the MUCL injury type (sport‐related or non‐sport‐related).

In conclusion, based on the evidence available in the selected peer reviewed published literature, both treatments obtained good results for most patients in daily and/or sport activities, although the complication rate was higher in the nonoperative management of MUCL injuries and their outcomes seem to be particularly reduced in high‐function‐demand patients. There is currently insufficient evidence in the literature to establish statistically significant differences in the effects of conservative versus surgical treatment on the functional outcomes of patients with these lesions. However, the ideal treatment should be chosen based on the expected functional requirement of every single patient (Fig. 2). Low‐function‐demand patients should be treated conservatively. However, even high‐function demand patients (for whom operative management is indicated) should be initially treated with a brief period of rehabilitation of at least 3 months. Whether residual elbow instability persists, surgery is indicated in both groups of patients (low‐demand and high‐demand), regardless of the MUCL injury type (sport‐related or non‐sport‐related). Finally, future trials should be developed to standardize the assessment of functional outcomes after different types of treatment, using standard and validated measures, to make a complete clinical and statistical comparison not only among the several treatment options but also among different types of patients.

## Supporting information


**Table S1** Detailed description of complications and outcomes.
