# Supplementary material for: Conservative Versus Surgical Management of Elbow Medial Ulnar Collateral Ligament Injury: A Systematic Review
Source: Orthop Surg. 2019 Nov 26;11(6):974–84. doi: 10.1111/os.12571 (PMC6904592; doi:10.1111/os.12571)
Supplement: Supplementary file 1 — Table S1 Detailed description of complications and outcomes. [file OS-11-974-s001.docx]

**Table 1. Detailed description of complications and outcomes.**

| **First Author (year)** | **No. of patients** | **Complications** | **Outcomes** |
| --- | --- | --- | --- |
| Iordens et al.  (2015) ^36^ | 27 | - 10 pts had fixator-related complication  - 7 pts required secondary surgery (5 pts elbow incongruency, 1 pt hardware defect), 1 pt pin tract fracture humerus (required plate fixation)  - 4 pts had a pin tract infection (2 required debridement, 2 oral antibiotics)  - 1 pt pin-tract fracture of ulna (conservative treatment)  - 1 pt had recurrent instability  - 13 pts heterotopic ossifications at 1 year | **DASH**  Mean score improved from 30 to 7  **MEPS**  Mean score increased from 80 to 100 |
| Kesmezacar et al. (2010) ^29^ | 21 | - 6 pts (28.6%) had neurological complaints (ulnar nerve neuropathy)  - 4 pts permanent varus-valgus instability  - 14 pts heterotopic ossification  - 81% of pts reported elbow stiffness, pain during sport activities, ROM limitation (only 4 pts, 19,1% reported a feeling of full recovery). | **MEPS**  Mean was 96.9  - Excellent in 20 pts  - Moderate in 1 pt  It considers only performance in daily life activity and not in challenging physical activities |
| Dines et al.  (2007) ^30^ | 22 | No intraoperative complications  Post-op complication: 4/22, 17% (3 re-operation)  - 1 postoperative ulnar neuritis that caused pain but no weakness  - 1 persistent pain and ulnar nerve symptoms (previous surgery and un transposition)  - 2 patients were treated for lysis of adhesions at a mean of 18 month | **Conway Scale**  -19/22 Excellent results  -2/22 Fair results  -1/22 Poor result |
| Dines et al.  (2008) ^31^ | 15 | No intraoperative complications  - 6 post-op complications: 1 required further surgery (1 pt retear), 2 post-operative stiffness,1 reactive synovitis, 1 ulnar neuritis, 1 medial epycondilitis | **Conway Scale** after 1st surgery  - 9 excellent results  - 2 good results  - 1 fair result  - 3 poor results  **Conway Scale** after 2nd surgery  - 5 excellent results  - 4 good results  - 2 fair results  - 4 poor results |
| Erickson et al.  (2016) ^27^ | 187 | - re-surgery in 5.3% (10/187) of pts, 7 of them had subsequent ulnar nerve transposition for persistent neurological symptoms | **Conway-Jobe scor**e 94.1% good/excellent while  4.3% fair.  **KJOC** was 90.4 ± 6.7  **Andrews-Timmerman** score was 92.5 ±7.1. |
| Osbahr et al.  (2010) ^28^ | 8 | -12.5% of pts returned to their previous level of play | **Conway Scale**  -1 excellent (12.5%)  -2 fair (25%)  -5 poor (62.5%) |
| Rhyou et al.  (2012) ^37^ | 29 | - no complications in group A (conservative treatment of torn MUCL without rupture  - no complications in group B (surgical repair of torn MUCL, rupture documented in MRI with valgus stress maneuvers) | **DASH**  Higher disability DASH score in group B compared with group A |
| Richard et al.  (2008) ^34^ | 11 | - 1 20° flexion contracture  - 3 acute ulnar nerve palsies with injury recovered after surgery | **DASH:** mean value 6 |
| Adolfsson et al.  (2017) ^26^ | 8 | - 1 pt ulnar nerve territory numbness | **Modified MEPS** |
| Chen et al.  (2017) ^18^ | 9 | - 1 pt mild discomfort extreme flexion and extension  - 2 pts ulnar neuropathy resolved within 3 months  - 1 pt permanent sensory disturbance to medial cutaneous nerve of the forearm  - 2 pts with preoperative ulnar nerve neuropathy recovered after surgery | **MEPS**  Mean MEPS 58 before surgery to mean MEPS 94 after surgery |
| Dines et al.  (2012) ^7^ | 10 | - no intra or post-operative complications | **Conway Scale**  - 9 excellent (90%)  - 1 fair (10%) |
| Jones et al.  (2014) ^32^ | 55 | - 4 postoperative complications in patients who developed ulnar neuritis after MUCL reconstruction  - 5 patients who did not achieve symmetric motion demonstrated extension deficits that were similar to their preoperative assessment. Notably, 4 of these patients (4/5, 80%) demonstrated advanced intra-articular disorders (capitellar OCD, n = 2; posteromedial olecranon osteophyte, n = 2) | **Conway Scale**  - 87.3% (48/55 pts) excellent results  - 3.6% (2/55 pts) good results  - 5.5% (3/55 pts) fair results  - 3.6% (2/55 pts) poor results  **Andrews-Timmerman score** 83.6 ± 7.2 (range, 30-100)  **KJOC** was 88.0 ± 6.0 (range, 40-100) |
| Kodde et al.  (2012) ^33^ | 20 | - 1 pt with ulnar nerve hyperesthesia  - 1 pt with a flexion contracture  - 1 pt with residual instability  (4 pts with preoperative ulnar nerve symptoms, 12 pts with traumatic MUCL rupture) | **Conway Scale**  - 18/20 Excellent result  - 2/20 Poor result  - **MEPS**  Preoperative 82 prs → 91pts |
| Podesta et al.  (2013) ^13^ | 34 | - No complications observed after PRP. Patients did experience variable degrees of post-injection inflammation at the injection site, including localized mild swelling that was controlled by moist heat application and analgesic pain medication for the first 24 hours after treatment.  - None of the patients developed post-procedure  infections, ulnar nerve irritation, or neuropathy | **DASH**  - Mean improvement from 21±16 to 1±6  **KJOC**  - Mean improvement from 46±15 to 93±7  - Average time to return to play 12 w (range, 10-15 w) |
| Savoie et al.  (2008) ^35^ | 60 | - 4 patients were considered to have failures  - 2 patients were considered to have a successful result but sustained late failure | **Carson score**  -The mean postoperative Andrews-Carson rating improved from 132 preoperatively to 188 postoperatively (*P* < 0.05)  - Excellent 85%; Good 8%; Fair 3.5%;Poor 3.5% |

**Pts**: *patients;* M**UCL**: *Medial Ulnar Collateral Ligament;* **DASH**: *Disability of* Arm, Shoulder and Hand; **MEPS:** *Mayo Elbow Performance;* **KJOC**: *Kerlan-Jobe Orthopaedic Clinic score.* **PRP*,*** *platelet-rich plasma*
